# Knowledge and Utilization of E-learning Platforms Among Medical Undergraduates in Haveri District, Karnataka State, India

**DOI:** 10.7759/cureus.88356

**Published:** 2025-07-20

**Authors:** Khundmeer Banu Athani, K S Govinda Swamy, Shivakumar Kerakkanavar, Hamida Kwas, Harish Rangareddy

**Affiliations:** 1 Physiology, Haveri Institute of Medical Sciences, Haveri, IND; 2 Biochemistry, Shimoga Institute of Medical Sciences, Shimoga, IND; 3 Orthopaedics, Haveri Institute of Medical Sciences, Haveri, IND; 4 Pulmonology, University of Sfax, Faculty of Medicine of Sfax, Gabès University Hospital, Gabès, TUN; 5 Biochemistry, Haveri Institute of Medical Sciences, Haveri, IND

**Keywords:** e-learning attitude, e-learning perceptions, online medical education, undergraduate medical student, undergraduate teaching strategies

## Abstract

Background

The digital transformation in medical education has led to a surge in the use of e-learning platforms. This study aimed to assess the knowledge, preferences, and barriers related to e-learning among medical undergraduates in Haveri, Karnataka.

Methods

A cross-sectional, questionnaire-based study was conducted with 111 medical undergraduate student respondents. Data collection included both quantitative (Likert-scale-based items) and qualitative (open-ended responses). Ten Likert-scale items (Q7-Q16) were used to explore the students’ ability to balance e-learning with traditional methods, perceived enhancement of knowledge, impact on academic performance, integration preferences, technological confidence, satisfaction with platforms, future intent to use, peer influence, customization of learning, and support for curricular integration. Quantitative data were analyzed using descriptive statistics, and qualitative responses were subjected to thematic analysis.

Results

Most students reported frequent (n = 48, 43.2%) or very frequent (n = 11, 9.9%) use of e-learning platforms, with video lectures being the most commonly accessed resource (n = 85, 76.6%). Platforms like YouTube (n = 49, 44.1%), Marrow (n = 20, 18%), Egurukul (n = 17, 15.3%), and others were commonly used. The credibility of resources was primarily judged based on faculty recommendations (n = 43, 38.7%) and platform reputation (n = 32, 28.8%). Likert-scale responses (Q7-Q16) showed that most students preferred integrating e-learning with traditional learning (Agree: n = 49, 44.1%; Strongly Agree: n = 18, 16.2%) and felt confident using technology (Agree: n = 51, 45.9%; Strongly Agree: n = 19, 17.1%). A majority agreed that e-learning resources enhanced their understanding (Agree: n = 52, 46.8%; Strongly Agree: n = 28, 25.2%) and improved academic performance (Agree: n = 52, 46.8%; Strongly Agree: n = 28, 25.2%). Thematic analysis revealed advantages such as flexibility, conceptual clarity, and self-paced learning. Reported disadvantages included screen-related health concerns, distractions, and reduced interactivity. Common barriers were poor internet connectivity (n = 49, 44.1%), cost of subscriptions (n = 39, 35.1%), and time constraints (n = 33, 29.7%).

Conclusion

E-learning is widely accepted among medical students in Haveri, though its optimal utilization faces technical and economic challenges. Incorporating structured e-learning modules into the medical curriculum, supported by appropriate access and guidance mechanisms, is recommended to enhance their effectiveness.

## Introduction

The integration of digital technologies into medical education has revolutionized the learning experience of students globally [1]. E-learning, characterized by its flexibility, visual richness, and learner-centric approach, has emerged as a prominent tool, especially in the context of the COVID pandemic and beyond globally [2]. In India, platforms such as Marrow and Prepladder have gained popularity among medical students for their tailored content and exam preparation resources accessed using smartphone technology [3]. Despite its widespread use, disparities in access, technical literacy, and institutional integration may affect the effectiveness of e-learning tools. This study aimed to comprehensively evaluate the knowledge, usage patterns, and perceived barriers associated with e-learning among medical undergraduate students in Haveri, Karnataka. Specifically, it sought to assess students’ awareness of various e-learning platforms, the frequency and context of their usage (e.g., for video lectures, exam preparation, or self-directed learning), and their perceptions regarding the effectiveness, accessibility, and integration of digital tools in the medical curriculum. Additionally, the study explored the challenges faced by students, such as technical limitations, platform credibility, and institutional support, in adopting e-learning as a regular component of their academic experience. Overall, this study evaluated the knowledge, usage patterns, and perceived barriers related to e-learning among medical undergraduates in Haveri, Karnataka.

## Materials and methods

Study design

This cross-sectional study was conducted at Haveri Institute of Medical Sciences, Karnataka, India, among all medical undergraduates except recently enrolled first-year MBBS students during the period from February 2025 to May 2025.

Study population and sampling

Students were briefed in person by the researchers about the study's purpose and procedures. Sample size (n) for the survey was calculated with the following equations: $x = Z \left( \frac{c}{100} \right)^2 r (100 - r)$ and $n = N \cdot \frac{x}{(N - 1) E^2 + x}$, where N is the population size, E is the margin of error (10%), r is the fraction of responses (50%), and $Z \left( \frac{c}{100} \right)$ is the critical value for confidence level c (5%) using the Raosoft calculator (Raosoft Inc., Seattle, Washington) [4]. A margin of error of 10% was initially assumed, and the sample size obtained was 96. All medical students were included through purposive sampling, and first-year students were excluded, as they were recently admitted to the program and exposure to e-learning in the context of medical education was lacking. A total of 298 participants were provided with the questionnaire, of which 111 responded, and the data were analysed. Ethical clearance for the study was obtained from the Institutional Ethics Committee of Haveri Institute of Medical Sciences (approval number: HIMS/IEC/2025/04).

Data collection tool

Data for the study were collected using a structured, self-administered questionnaire that had been validated by faculty experts. The instrument comprised three sections: Section A gathered demographic information (age, gender) along with usage patterns and general perceptions of e-learning; Section B included Likert-scale items assessing knowledge, utilization, and perceptions regarding e-learning platforms; and Section C consisted of open-ended questions aimed at exploring the perceived advantages, disadvantages, and barriers to e-learning (Appendices). To assess the reliability of the questionnaire, internal consistency was evaluated using Cronbach’s alpha, which yielded a value of 0.89, indicating high reliability.

Data analysis

Quantitative data were analyzed using SPSS version 16 (SPSS Inc., Chicago, IL, USA) to generate frequency distributions, percentages, and non-parametric tests. Thematic analysis was employed for qualitative data using an inductive coding approach and performed manually due to the manageable volume.

## Results

A total of 111 undergraduate medical students participated in the study, all aged between 18 and 25 years. The gender distribution was nearly equal, with 56 males (50.5%) and 55 females (49.5%). Regarding platform usage, YouTube emerged as the most commonly used e-learning resource (n = 49, 44.1%), followed by Marrow (n = 20, 18%), Egurukul (n = 17, 15.3%), and other platforms, including ChatGPT and PW MedEd (n = 25, 22.6%). The frequency of e-learning utilization was reported as “frequent” by 48 students (43.2%) and “occasional” by 38 students (34.2%), with 11 students (9.9%) indicating that they “always” used such resources. A minority used them “rarely” (n = 12, 10.8%) or “never” (n = 2, 1.8%). Video lectures were the predominant type of content accessed (n = 85, 76.6%), while fewer students used interactive case studies (n = 8, 7.2%) or textbooks and quizzes (n = 18, 16.2%). In evaluating the credibility of e-learning resources, 43 students (38.7%) relied on faculty recommendations, 32 (28.8%) on platform reputation, and 23 (20.7%) on trusted medical sources; 5 respondents (4.5%) were unsure how to assess credibility. The demographic characteristics and utilization patterns have been depicted in Table 1.

**Table 1:** Participant demographic characteristics and behavioral patterns regarding e-learning The data represent the participant demographic characteristics and behavioral patterns regarding e-learning, and the values are expressed as frequencies (n) and corresponding percentages (%).

Q. No.	Item	Categories	n (%)
Q1	Age	18–25 years	111 (100%)
Q2	Gender	Male	56 (50.5%)
		Female	55 (49.5%)
Q3	Most Used Platforms	YouTube	49 (44.1%)
		Marrow	20 (18%)
		Egurukul	17 (15.3%)
		Others (viz., ChatGPT, PW MedEd)	25 (22.6%)
Q4	Frequency of Use	Always	11 (9.9%)
		Frequently	48 (43.2%)
		Occasionally	38 (34.2%)
		Rarely	12 (10.8%)
		Never	2 (1.8%)
Q5	Type of Resource	Video lectures	85 (76.6%)
		Case studies	8 (7.2%)
		Textbooks/Quizzes	18 (16.2%)
Q6	Credibility Assessment	Faculty recommendation	43 (38.7%)
		Trusted sources	23 (20.7%)
		Reputation	32 (28.8%)
		Not sure	5 (4.5%)

Responses to Likert-scale items (Q7-Q16), as shown in Table 2, revealed that 52 students (46.8%) agreed and 28 (25.2%) strongly agreed that e-learning enhanced their academic performance. Similarly, a majority either agreed (n = 52, 46.8%) or strongly agreed (n = 28, 25.2%) that e-learning improved their understanding and knowledge. Most students expressed confidence in using technology for e-learning, with 51 (45.9%) agreeing and 19 (17.1%) strongly agreeing. Preference for integrating e-learning with traditional methods was evident, with 49 students (44.1%) agreeing and 18 (16.2%) strongly agreeing. Notably, 57 participants (51.4%) agreed that e-learning enabled customized learning experiences. A strong inclination toward incorporating e-learning into the formal medical curriculum was also observed, with 48 (43.2%) agreeing and 19 (17.1%) strongly agreeing.

**Table 2:** Distribution of responses to Likert-scale items on knowledge, utilization, and perceptions regarding e-learning platforms The data represent the distribution of responses on Likert-scale items assessing knowledge, utilization, and perceptions regarding e-learning platform (Scale: 1 = Strongly Disagree, 2 = Disagree, 3 = Neutral, 4 = Agree, 5 = Strongly Agree) and values are expressed as frequencies (n) and corresponding percentages (%).

Q. No.	Item	Strongly disagree n (%)	Disagree n (%)	Neutral n (%)	Agree n (%)	Strongly Agree n (%)
Q7	I effectively balance e-learning with traditional methods	4 (3.6%)	3 (2.7%)	46 (41.4%)	47 (42.3%)	11 (9.9%)
Q8	E-learning enhances understanding and knowledge	4 (3.6%)	1 (0.9%)	26 (23.4%)	52 (46.8%)	28 (25.2%)
Q9	E-learning positively impacts academic performance	3 (2.7%)	—	28 (25.2%)	52 (46.8%)	28 (25.2%)
Q10	I prefer integrating e-learning with traditional methods	2 (1.8%)	2 (1.8%)	40 (36.0%)	49 (44.1%)	18 (16.2%)
Q11	I feel confident using technology for e-learning	2 (1.8%)	1 (0.9%)	38 (34.2%)	51 (45.9%)	19 (17.1%)
Q12	I am satisfied with available e-learning platforms	1 (0.9%)	6 (5.4%)	38 (34.2%)	52 (46.8%)	14 (12.6%)
Q13	I want to increase use of e-learning in future	2 (1.8%)	3 (2.7%)	42 (37.8%)	48 (43.2%)	16 (14.4%)
Q14	Peer influence affects e-learning usage	2 (1.8%)	12 (10.8%)	46 (41.4%)	40 (36.0%)	11 (9.9%)
Q15	E-learning helps customize learning to my needs	3 (2.7%)	2 (1.8%)	33 (29.7%)	57 (51.4%)	16 (14.4%)
Q16	E-learning should be integrated more into the curriculum	2 (1.8%)	4 (3.6%)	38 (34.2%)	48 (43.2%)	19 (17.1%)

Responses to the 10 Likert-scale items assessing perceptions of e-learning (Q7-Q16) were analyzed and grouped under thematic categories for interpretive clarity as shown in Table 3.

**Table 3:** Descriptive statistics for Likert scale items on e-learning categorized thematically (n = 111) Responses were recorded on a 5-point Likert scale (1 = Strongly Disagree, 5 = Strongly Agree). All items had complete responses (n = 111).

Thematic Category	Item No.	Survey Statement	Mean ± SD	Min	Max
Perceptions of E-learning Effectiveness	Q8	E-learning enhances understanding and knowledge	3.89 ± 0.92	1	5
	Q9	E-learning positively impacts academic performance	3.92 ± 0.86	1	5
	Q15	E-learning helps customize learning to my needs	3.73 ± 0.83	1	5
Attitudes and Preferences	Q7	I effectively balance e-learning with traditional methods	3.52 ± 0.85	1	5
	Q10	I prefer integrating e-learning with traditional methods	3.71 ± 0.82	1	5
	Q13	I want to increase the use of e-learning in the future	3.66 ± 0.83	1	5
Technology Confidence and Satisfaction	Q11	I feel confident using technology for e-learning	3.76 ± 0.81	1	5
	Q12	I am satisfied with the available e-learning platforms	3.65 ± 0.81	1	5
Social and Curricular Perspectives	Q14	Peer influence affects e-learning usage	3.41 ± 0.88	1	5
	Q16	E-learning should be integrated more into the curriculum	3.70 ± 0.86	1	5

Under "Perceptions of E-learning Effectiveness", students reported that e-learning enhanced their understanding and knowledge (Q8, Mean = 3.89, SD = 0.92) and positively impacted academic performance (Q9, Mean = 3.92, SD = 0.86), the highest among all items. Additionally, e-learning was perceived to aid in customized learning (Q15, Mean = 3.73, SD = 0.83), underscoring its potential to support learner-specific needs. In the category "Attitudes and Preferences", respondents expressed moderate agreement with statements indicating effective balancing of e-learning with traditional methods (Q7, Mean = 3.52, SD = 0.85), a preference for integration of modalities (Q10, Mean = 3.71, SD = 0.82), and a willingness to increase future use of e-learning (Q13, Mean = 3.66, SD = 0.83). Regarding "Technology Confidence and Satisfaction", students generally felt confident in using technology for e-learning (Q11, Mean = 3.76, SD = 0.81) and were reasonably satisfied with the available platforms (Q12, Mean = 3.65, SD = 0.81), indicating adequate digital literacy and access. Under "Social and Curricular Perspectives", peer influence was rated comparatively lower (Q14, Mean = 3.41, SD = 0.88), suggesting it may play a modest role in shaping e-learning behaviors. Notably, respondents showed support for deeper integration of e-learning into the formal curriculum (Q16, Mean = 3.70, SD = 0.86), reflecting a favorable attitude toward institutional adoption of blended learning models. Overall, the findings reflect a predominantly positive perception of e-learning across multiple domains, with the highest ratings attributed to its perceived impact on academic performance and knowledge enhancement.

Spearman’s correlation analysis revealed significant positive associations among the Likert-scale items (Q7-Q16), indicating consistent patterns in students’ perceptions of e-learning. Notably, balancing e-learning with traditional methods (Q7) was moderately correlated with enhanced understanding (Q8, rho = 0.513, p < 0.01) and improved academic performance (Q9, rho = 0.478, p < 0.01). Confidence in using technology (Q11) was strongly linked to satisfaction with platforms (Q12, rho = 0.655, p < 0.01), learning customization (Q15, rho = 0.700, p < 0.01), and support for curricular integration (Q16, rho = 0.517, p < 0.01). The strongest correlation was observed between perceived enhancement of understanding and academic performance (Q8-Q9, rho = 0.768, p < 0.01). Intent to use e-learning in the future (Q13) correlated well with academic benefits and personalization. Peer influence (Q14) showed weaker correlations across items. These findings suggest a coherent structure in e-learning perceptions, with academic value, technological ease, and customization emerging as central, interrelated themes as depicted in Table 4.

**Table 4:** Spearman’s correlation matrix for Likert scale items (Q7–Q16) Spearman’s rho was used to assess pairwise correlations between items. Correlation is significant at the 0.01 level (**) and 0.05 level () (2-tailed)*. Diagonal values (1.000) represent perfect self-correlation and have been shown only once for clarity.

	Q7	Q8	Q9	Q10	Q11	Q12	Q13	Q14	Q15	Q16
Q7	1.000	0.513**	0.478**	0.445**	0.407**	0.327**	0.155	0.327**	0.409**	0.273**
Q8	0.513**	1.000	0.768**	0.537**	0.543**	0.470**	0.508**	0.311**	0.683**	0.536**
Q9	0.478**	0.768**	1.000	0.591**	0.565**	0.503**	0.537**	0.253**	0.689**	0.525**
Q10	0.445**	0.537**	0.591**	1.000	0.528**	0.419**	0.213*	0.302**	0.467**	0.389**
Q11	0.407**	0.543**	0.565**	0.528**	1.000	0.655**	0.485**	0.291**	0.700**	0.517**
Q12	0.327**	0.470**	0.503**	0.419**	0.655**	1.000	0.449**	0.246**	0.533**	0.430**
Q13	0.155	0.508**	0.537**	0.213*	0.485**	0.449**	1.000	0.297**	0.626**	0.623**
Q14	0.327**	0.311**	0.253**	0.302**	0.291**	0.246**	0.297**	1.000	0.340**	0.399**
Q15	0.409**	0.683**	0.689**	0.467**	0.700**	0.533**	0.626**	0.340**	1.000	0.585**
Q16	0.273**	0.536**	0.525**	0.389**	0.517**	0.430**	0.623**	0.399**	0.585**	1.000

The distribution of responses to questions Q7 through Q16 was assessed further for association with gender using Pearson's chi-squared tests of independence. Across the majority of questions (Q7-Q11, Q13-Q16), no statistically significant differences were observed between female and male participants in response patterns (all p > 0.05), indicating comparable gender-based perceptions or attitudes for these items as shown in Table 5.

**Table 5:** Gender-wise distribution of responses and Pearson chi-square test results for Likert scale questions 7 to 16 *Significant at p < 0.05

Q. No.	Gender	Response 1	Response 2	Response 3	Response 4	Response 5	Total	Pearson Chi-Square (df=4)	p-value
Q7	Female	1	0	28	21	5	55	6.788	0.148
	Male	3	3	18	26	6	56		
Q8	Female	1	0	16	23	15	55	4.211	0.378
	Male	3	1	10	29	13	56		
Q9	Female	0	15	24	16	—	55	4.013	0.260
	Male	3	13	28	12	—	56		
Q10	Female	1	0	20	23	11	55	3.064	0.547
	Male	1	2	20	26	7	56		
Q11	Female	1	0	22	23	9	55	2.481	0.648
	Male	1	1	16	28	10	56		
Q12	Female	0	4	27	17	7	55	14.626	0.006*
	Male	1	2	11	35	7	56		
Q13	Female	0	0	20	27	8	55	5.837	0.212
	Male	2	3	22	21	8	56		
Q14	Female	0	7	26	16	6	55	4.798	0.309
	Male	2	5	20	24	5	56		
Q15	Female	1	1	18	28	7	55	0.865	0.930
	Male	2	1	15	29	9	56		
Q16	Female	0	1	21	24	9	55	3.465	0.483
	Male	2	3	17	24	10	56		

Notably, responses to Question 12, which assessed participants’ satisfaction with the currently available e-learning platforms, revealed a statistically significant difference between female and male respondents (χ²(4) = 14.626, p = 0.006). Females and males showed distinct patterns in their satisfaction levels. Specifically, a higher proportion of female participants selected moderate satisfaction categories (responses 3 and 4), while male participants more frequently endorsed higher satisfaction (response 4) compared to females. The significant positive correlations observed between the gender and response categories indicate that gender is moderately associated with how satisfied respondents felt about the e-learning platforms. This gender disparity in satisfaction could reflect varied user experiences, preferences, or accessibility issues related to e-learning tools. Possible factors influencing this difference include differences in technological familiarity, the quality or relevance of content accessed, or the adaptability of platforms to individual learning styles. Such findings underscore the need for further qualitative investigations to explore barriers and facilitators of e-learning satisfaction among different gender groups.

Thematic analysis of open-ended responses identified three major domains: advantages, disadvantages, and barriers, as shown in Table 6.

**Table 6:** Thematic analysis of open-ended questions on perceived advantages, disadvantages, and barriers to e-learning The table presents the results of a thematic analysis conducted on open-ended responses regarding participants' perceived advantages, disadvantages, and barriers to e-learning. Themes were identified through qualitative coding, and the number of participants (n) endorsing each theme is reported alongside corresponding percentages (%).

Q. No.	Theme	Sub theme	Student perceptions	n (%)
Q17	Advantages	Flexibility & Accessibility	“Can learn anytime, anywhere.”	76 (68.5)
		Conceptual Clarity	“Helps in better understanding.”	68 (61.3)
		Personalized Learning	“Can watch at own pace, replay.”	41 (36.9)
Q18	Disadvantages	Eye Strain & Health	“Eye pain due to long screen time.”	47 (42.3)
		Distractions	“Distracted by mobile notifications.”	53 (47.7)
		Lack of Interaction	“No direct doubt clarification.”	38 (34.2)
Q19	Barriers	Poor Network	“Internet connectivity is slow.”	49 (44.1)
		Cost of Platforms	“Subscriptions are expensive.”	39 (35.1)
		Time Constraints	“Hard to balance with college studies.”	33 (29.7)

Among advantages, flexibility and accessibility were most frequently cited (n = 76, 68.5%), followed by improved conceptual clarity (n = 68, 61.3%) and personalized learning opportunities (n = 41, 36.9%). Disadvantages primarily included distractions from mobile devices and social media (n = 53, 47.7%), eye strain or other health-related concerns due to prolonged screen time (n = 47, 42.3%), and a lack of real-time interaction with faculty or peers (n = 38, 34.2%). Reported barriers to effective e-learning utilization included poor internet connectivity (n = 49, 44.1%), high costs of platform subscriptions (n = 39, 35.1%), and time constraints due to academic workload (n = 33, 29.7%).

## Discussion

This study provides valuable insights into the patterns of e-learning utilization, perceptions, and challenges among medical undergraduates in Haveri, Karnataka. The findings reveal that e-learning is widely embraced, with YouTube (n = 49, 44.1%), Marrow (n = 20, 18%), and Egurukul (n = 17, 15.3%) being the most commonly used platforms. Video lectures were the predominant resource accessed (n = 85, 76.6%), reflecting students' preference for audiovisual learning modalities, which is consistent with global trends in digital pedagogy. A substantial proportion of students reported using e-learning frequently (n = 48, 43.2%) or occasionally (n = 38, 34.2%), and many perceived these platforms as enhancing their academic performance and understanding of complex medical content.

The educational value of platforms such as YouTube has been increasingly documented. Desai et al. demonstrated that a nephrology-focused YouTube channel was well-received by healthcare professionals, with 81.0% finding the content useful, 85.0% deeming it accurate, and 83.0% judging it objective and current [5], suggesting that when educational material is hosted on a credible, well-curated platform, it is more likely to be trusted, positively received, and considered reliable by its target audience. Similarly, Garside et al. found that their YouTube-hosted Mini Geriatric E-Learning Modules improved learners’ confidence in managing geriatric cases, owing to the concise and targeted nature of the videos [6]. This underscores the importance of credibility in enhancing the educational value and impact of digital learning resources in medical education. In the present study, YouTube’s popularity further validates its role as an effective adjunct for self-directed learning among Indian medical students. 

A recent comparative study by Liu et al. on the quality of videos related to laryngeal carcinoma across YouTube, Bilibili, and TikTok revealed that while TikTok had better audience engagement, YouTube demonstrated the highest overall quality based on the Patient Education Materials Assessment Tool (PEMAT), Video Information and Quality Index (VIQI), Global Quality Score (GQS), and modified DISCERN (mDISCERN) [7]. Their findings highlight the importance of video source credibility, with professional authors significantly outperforming non-professionals in terms of content accuracy and educational value [7]. This aligns with our data, where 43 students (38.7%) trusted faculty recommendations when assessing resource credibility, and 32 (28.8%) relied on the reputation of the platform. Furthermore, AlBloushi et al.'s evaluation of 465 YouTube videos on diabetic retinopathy revealed that 72.5% were educationally valuable, especially those uploaded by hospitals or containing animated content [8]. These findings echo the present study’s results, suggesting that institutional and professionally curated content is both trusted and widely consumed by learners.

The findings of our study, in which a majority of students (n = 52, 46.8%) agreed and n = 28 (25.2%) strongly agreed, reported that e-learning improved their academic performance, which are consistent with emerging global evidence highlighting the pedagogical benefits of digital learning environments. Several studies across different educational settings and student populations reinforce the claim that e-learning, when well-implemented, not only enhances learner satisfaction but also improves measurable academic outcomes. For instance, Alenezi et al. evaluated the transition of a psychiatry course from onsite to online learning during the COVID-19 pandemic and observed a statistically significant improvement in students’ grades during the online phase as compared to the traditional mode, alongside increased satisfaction with course organization and learning resources [9]. Similarly, in Ghana, Bossman et al. found that student satisfaction with e-learning platforms significantly mediated the relationship between system quality and academic performance. Factors such as course quality, instructor competence, and ease of platform use directly contributed to learning gains [10]. These findings support the notion that satisfaction is not merely a by-product of user experience but a pivotal determinant of academic success in digital environments.

Further supporting this, Rezaee et al. demonstrated that the application of case-based e-learning (CBEL) in nursing education led to statistically significant improvements in both academic performance and problem-solving abilities (p < 0.001), emphasizing the role of interactivity and contextual learning in student outcomes [11]. This resonates with our finding that 51.4% of students felt e-learning helped them customize learning to their needs - a core feature of CBEL and similar active learning models. A unique contribution comes from Nebot-Cegarra et al., who conducted a comparative analysis of blended learning in human anatomy education. Their large, homogeneous sample (n = 1160) revealed that while top-performing students slightly favored face-to-face modules, lower-performing students demonstrated improved outcomes through e-learning. Notably, the correlation between attempted responses and academic performance was strong across both modalities, particularly in e-learning for the lower quartiles [12]. This finding aligns with our observation that students with varied learning styles benefit from the self-paced nature of e-resources like video lectures, which were preferred by 85 students (76.6%) in our study. Building upon these foundations, the integration of Artificial Intelligence (AI) into e-learning presents a transformative opportunity for performance optimization. Mahafdah et al. demonstrated that AI tools, particularly deep learning models, such as convolutional neural networks (CNNs) and recurrent neural networks (RNNs), can accurately predict student performance, identify learning gaps, and adapt content delivery in real time [13]. These findings herald the next phase of e-learning evolution, where content is not only accessible and interactive but also dynamically responsive to learner behavior and cognitive patterns. However, it is important to note that our study did not incorporate AI-based tools, such as ChatGPT or adaptive learning algorithms, in data collection, analysis, or intervention design. The findings of our study are therefore based solely on participant self-reports and conventional statistical methods, without AI-driven enhancements.

In our study, thematic analysis of open-ended responses revealed that the most frequently cited advantage of e-learning was its flexibility and accessibility (n = 76, 68.5%), followed by improved conceptual clarity (n = 68, 61.3%) and opportunities for personalized learning (n = 41, 36.9%). The ability to learn "anytime, anywhere" makes e-learning a particularly attractive modality for medical students facing intense academic workloads and variable schedules. This finding echoes the conclusions of Chandrashekar et al., who advocated for a self-paced, integrated e-learning module on tobacco counseling for medical and dental students in India. Their mixed-methods study demonstrated that time constraints within the conventional curriculum made traditional delivery of tobacco counseling training unfeasible, and participants emphasized the need for asynchronous, flexible learning formats to fill this curricular gap [14].

Similarly, in a structured intervention study, Vaziri et al. developed case-based, interactive pediatric pulmonology modules that were made accessible to students and residents during their clinical rotations. The modules were positively received, and post-test scores improved by up to 21%, indicating that self-paced, virtual learning enhances both knowledge retention and clinical decision-making. Notably, trainees reported that the modules helped clarify concepts they had previously found difficult during the rotation, thereby improving their learning outcomes [15]. In our cohort, a significant proportion of students (n = 68, 61.3%) also cited improved conceptual clarity as a benefit of e-learning. This aligns with broader pedagogical evidence that multimedia tools, including video lectures and interactive cases, facilitate dual-channel processing of information (visual and auditory), which strengthens understanding of complex subjects.

Furthermore, 36.9% (n = 41) of students in our study appreciated the opportunity for personalized learning, which refers to adjusting pace, revisiting content, and selecting preferred formats. This advantage is supported by the experience of Daudelin et al., who developed a self-paced online nutrition science course based on dissemination and implementation (D&I) frameworks. Their efforts led to sustained increases in participation and strong feedback from both learners and instructors, who found that the content filled critical knowledge gaps while allowing flexibility in how it was consumed. The personalized, adaptable nature of the modules was key to their acceptability and success [16]. Collectively, these findings reinforce the idea that e-learning not only complements traditional instruction but also accommodates diverse learning needs, especially in rigorous medical education environments. The flexibility and conceptual support provided by digital modules can play a crucial role in enhancing the overall educational experience when thoughtfully designed and implemented.

Nevertheless, the study also brought to light notable disadvantages. Distractions from mobile notifications and social media were reported by 53 students (47.7%), while 47 students (42.3%) cited eye strain and other health-related concerns due to extended screen time. Additionally, 38 students (34.2%) noted a lack of direct interaction with instructors as a key limitation, echoing concerns about the reduced interpersonal engagement inherent in many asynchronous e-learning models. Chen et al. offered an additional perspective by identifying specific psychological and behavioral correlates of academic performance in e-learning among medical technology students. Their data indicated that motivation, study strategies, and burnout levels vary significantly across performance groups, and that high performers often experienced emotional exhaustion despite their achievements [17]. These findings underline the importance of tailoring e-learning interventions to support student well-being and individual learning styles, a factor that may help explain the demand for more personalized learning pathways, as expressed by students in our open-ended responses.

Despite the numerous benefits of e-learning, our findings highlight key barriers that continue to hinder its optimal utilization. Among participants, the most commonly cited obstacle was poor network connectivity (n = 49, 44.1%), followed by high internet subscription costs (n = 39, 35.1%) and time constraints due to academic workload (n = 33, 29.7%). These challenges reflect the ongoing digital divide, particularly in low- and middle-income settings, where infrastructure and affordability continue to limit equitable access to digital learning platforms. Similar trends have been observed in other studies. For instance, Kumar et al., using a Strengths, Opportunities, Aspirations, Results (SOAR) analysis framework among undergraduate medical and dental students in India, identified internet connectivity issues as a significant barrier to e-learning adoption, alongside eye strain, distractions, and a preference for conventional classroom teaching. These findings underscore the importance of reliable digital infrastructure and learner adaptability in maximizing the effectiveness of e-modules [18].

Moreover, a study from Pakistan by Abbas et al. found that 77% of preclinical medical students faced technical difficulties such as unstable internet access and limited IT skills, with 81% expressing overall dissatisfaction with their first online learning experience. A substantial proportion also reported dissatisfaction with institutional readiness, citing a lack of interaction and suboptimal course structure. These systemic limitations point to the need for not just technological upgrades but also faculty development and pedagogical restructuring in online education delivery [19]. In geographically dispersed and economically constrained regions like the Pacific Islands, Reddy et al. examined tablet-based mobile learning in higher education. While students expressed willingness to embrace tablet learning, they emphasized the need for prior training and better digital preparedness. Even in contexts where devices were available, inconsistent access to broadband and varying levels of digital literacy remained major hurdles. Their study emphasized that readiness, perception, and institutional support must align to ensure the successful implementation of technology-enabled education [20].

Taken together, these findings, along with our own, suggest that while e-learning offers significant educational advantages, its effectiveness is contingent upon the technological, economic, and logistical ecosystem in which learners are situated. To bridge the digital divide, academic institutions must invest in infrastructure, affordable data plans, and flexible scheduling, while also supporting both faculty and students through training and blended learning models. Importantly, 48 students (43.2%) agreed and 19 (17.1%) strongly agreed that e-learning should be more fully integrated into the medical curriculum. This consensus (n = 67, 60.3%) supports the development of blended learning models that harmonize online and in-person educational strategies, an approach particularly suited to the evolving needs of medical students in India.

Limitations

The findings should be interpreted within the context of certain limitations. First, this was a single-center study, which may limit generalizability to other institutions with different academic environments or digital infrastructure. Second, data were self-reported, introducing the possibility of response bias and recall inaccuracies. Third, while the open-ended responses provided rich qualitative insights, the thematic analysis was exploratory and done manually. The absence of inter-coder reliability testing for the qualitative thematic analysis has to be acknowledged as a limitation. Although themes were identified systematically, the consistency of coding was not formally validated. Future studies shall employ software such as KH coder for free text extraction and NVivo for structured coding, conducting peer debriefing to minimize interpretive bias, and calculating inter-coder agreement using statistical measures like Cohen’s kappa. The study did not assess objective academic performance data, which could have strengthened the association between e-learning usage and educational outcomes. Another limitation of the study is the modest response rate (~37%), which may raise concerns regarding potential non-response bias, as participants who chose to respond might differ systematically from non-respondents in their perceptions and usage of e-learning. However, it is important to note that the final sample size of 111 exceeded the calculated minimum required sample size of 96, thereby ensuring adequate statistical power and preserving the validity and reliability of the analysis. This strengthens the internal consistency of the study outcomes despite the lower response proportion.

## Conclusions

This study highlights the widespread acceptance and perceived benefits of e-learning among medical undergraduates in Haveri. Students demonstrated a clear preference for flexible, video-based learning resources that complement traditional instruction. While e-learning was associated with enhanced understanding and academic confidence, challenges such as distractions, screen-related health issues, high costs, and limited network access remain significant barriers. To maximize the benefits of e-learning, medical institutions must focus on integrating digital resources into the formal curriculum, improving infrastructure, and providing guidance to optimize student engagement. Future multi-institutional studies incorporating academic outcome measures are warranted to further evaluate the effectiveness of blended learning strategies in Indian medical education.

## Appendices

**Table 7:** Study questionnaire

Item	Response
1. Age	_______
2. Gender	☐ Male ☐ Female ☐ Prefer not to say ☐ Other: ___________
3. Which e-learning platforms or websites have you used for medical education?	___________
4. How often do you utilize e-learning resources specifically designed for medical education?	☐ Never ☐ Rarely ☐ Occasionally ☐ Frequently ☐ Always
5. What types of resources do you typically access through medical e-learning platforms? (Select all that apply)	☐ Video lectures ☐ Interactive Case studies ☐ Medical textbooks and references ☐ Online quizzes and practice questions ☐ Others (please specify): ___________
6. How do you assess the credibility and reliability of the medical e-learning resources you use?	a) Rely on recommendations from professors or colleagues b) Check the credentials and qualifications of content creators c) Consider reputation and credibility of the platform or website d) Verify information through trusted medical sources e) Not sure how to assess credibility and reliability

Item	Strongly Disagree (1)	Disagree (2)	Neutral (3)	Agree (4)	Strongly Agree (5)
7. I effectively balance e-learning with traditional methods	☐	☐	☐	☐	☐
8. E-learning enhances understanding and knowledge	☐	☐	☐	☐	☐
9. E-learning positively impacts academic performance	☐	☐	☐	☐	☐
10. I prefer integrating e-learning with traditional methods	☐	☐	☐	☐	☐
11. I feel confident using technology for e-learning	☐	☐	☐	☐	☐
12. I am satisfied with available e-learning platforms	☐	☐	☐	☐	☐
13. I want to increase use of e-learning in future	☐	☐	☐	☐	☐
14. Peer influence affects e-learning usage	☐	☐	☐	☐	☐
15. E-learning helps customize learning to my needs	☐	☐	☐	☐	☐
16. E-learning should be integrated more into the curriculum	☐	☐	☐	☐	☐

Question	Response
17. What are the advantages of e-learning in medical education?	____________________________________________
18. What are the disadvantages of e-learning in medical education?	____________________________________________
19. What do you think are the barriers to effective utilization of e-learning resources?	____________________________________________
